# Landscape-scale terrestrial factors are also vital in shaping Odonata assemblages of watercourses

**DOI:** 10.1038/s41598-019-54628-7

**Published:** 2019-12-03

**Authors:** H. Beáta Nagy, Zoltán László, Flóra Szabó, Lilla Szőcs, György Dévai, Béla Tóthmérész

**Affiliations:** 1 MTA-DE Biodiversity and Ecosystem Services Research Group, Debrecen, Egyetem sq. 1, H-4032 Hungary; 2 Hungarian Department of Biology and Ecology, Babeş-Bolyai University, str. Clinicilor nr. 5–7, 400006 Cluj-Napoca, Romania; 3 Department of Hydrobiology, University of Debrecen, Debrecen, Egyetem sq. 1, H-4032 Hungary; 4 Ecology Department, University of Debrecen, Debrecen, Egyetem sq. 1, H-4032 Hungary

**Keywords:** Community ecology, Conservation biology

## Abstract

Habitat loss and fragmentation causes a decline in insect populations. Odonata (both dragonflies and damselflies) are especially threatened by the destruction of both aquatic and terrestrial environment. Moreover, effects of large-scale habitat heterogeneity on Odonata assemblages are poorly studied. In a two years study along East-European lowland watercourses both aquatic and terrestrial environment were studied to reveal the importance of local (e.g. water depth, macrovegetation cover, etc.) and landscape-scale (e.g. farmland patch size, forest patch proportion, etc.) variables to Odonata (as well as to dragonflies and damselflies separately) through increasing spatial sampling scales. The specimens were sampled using 500 m long transects from May to September. Results, both on local and landscape scales emphasized the importance of terrestrial environment on Odonata. Local variables influence damselflies, while dragonflies are more sensitive to landscape variables. Damselfly’s diversity decreased with increasing macrovegetation cover, while dragonfly’s diversity decreased with the increasing degree of land use intensification, but increased with the length of watercourses. It is thus vital to stress the importance of partial watercourse clearing, and moderate maintenance of traditional farm management based on small parcel farming near watercourses to maintain diverse and healthy Odonata assemblages.

## Introduction

Odonata are real flagship taxa of freshwater ecosystems, and often used as indicator species to assess the quality of their close environment^1^. Their high diversity, complex life history, rapid development and their essential role in food webs^2,3^ make them ideal model insects for ecological surveys. Healthy aquatic habitats are crucial for the development of Odonata; beside this, adults also need resource-rich terrestrial habitats for maturation, feeding, resting, and mating^4^. Furthermore, Odonata are also sensitive to the landscape composition and configuration; their sensitivity to landscape quality can even exceed those of water hydrography and chemistry or other local ecological parameters describing the aquatic environment^5^. The presence and abundance of Odonata along watercourses are also affected by several conditions like water quality^6^, competition between larvae^7^, competition between adults^8^, dispersal ability of adults^9^, and the surrounding landscape^10^.

Human-caused habitat loss and fragmentation has become the major threatening factor during the last few decades for several taxa^11^. While several studies explore the influence of habitat loss on terrestrial populations and communities^12–14^, relatively few studies focus on the relationship between landscape changes and aquatic invertebrates such as Odonata. However, a remarkable rise in the number of studies regarding terrestrial effects on Odonata communities have emerged during the last ten years^10,15–17^.

The literature on Odonata-environment relationship is largely restricted to single or few species, and usually consider only a few landscape variables. The majority of the existing studies are focusing on the influence of bankside and riparian vegetation, analysing the presence of buffer strips or the extent of shading canopy^1,18–20^. Other studies address the relationship of Odonata and forests, underlying the importance of trees and shrubs for these insects^16,21,22^. Another group of studies reveal the major importance of connectivity between water bodies for Odonata^15,23–25^. Only a small number of studies targets Odonata assemblages using both local and landscape variables as predictors to understand their occurrence, abundance and community structure^4,10,26^.

The purpose of the study was to explore the effect of local (i.e. aquatic) and landscape variables (length of watercourses, forest patch proportion, and farmland patch size) on Odonata assemblages along lowland watercourses in two Central-Eastern European countries. Considering both the features of local habitat, and the surrounding landscape, the variables that were essential for the maintenance of rich Odonata assemblages were identified. The importance of local and landscape variables was also assessed by considering the two major Odonata groups separately (Zygoptera and Anisoptera) to explore taxa-specific sensitivities to the studied variables. The aim of this study was to conclude: (i) Which local biotic variables affect Odonata species diversity? (ii) Which landscape variables affect Odonata species diversity? (iii) Is there any difference in the sensitivity of the two suborders regarding the local and landscape variables?

## Results

Over the two-year period the number of specimens counted was 10,884 belonging to 34 species (Supplementary Material, Table S1). The Zygoptera and Anisoptera abundance showed no significant difference between years (χ^2^ = 3.54, df = 1, p = 0.06, Table 1) whereas the Zygoptera (χ^2^ = 1336.2, df = 10, p < 0.001) and Anisoptera (χ^2^ = 1077.5, df = 10, p < 0.001) showed significant differences (site-specific mean abundances).

**Table 1:** Number of observed species and the number of individuals (specimens).

Year/group	Zygoptera	Anisoptera	Zygoptera	Anisoptera
	species		specimens	
2015	14	18	2590	2642
2016	13	17	2901	2751
Total	15	19	5491	5393

### Local biotic variables

The water depth varied between 0.2 and 1.0 meter, with an average of 0.6 meter (±0.2). The width of watercourses varied between 1.9 and 10.4 meter, with an average of 4.2 meter (±2.2). The watercourses were found to have a relatively high-water surface macrovegetation cover: it varied from 6% to 95%, with an average of 72% (±27%). Eight sites out of eleven had higher than 75% vegetation cover, and only one had a lower than 10%. The percentage of banksides tree cover varied between 1.6 and 65% with an average of 37% (±23%). The average bankside cover of herbs was relatively high: 70% (±18), and it varied between 41% and 98%. The average plant height of the banksides was 60 cm (±23), varying between 24−93 cm.

Significant negative correlation was found between the percentage of water surface macrovegetation cover and Odonata diversity: with increasing surface cover the diversity of Odonata decreased (Table 2). This correlation was found to be significant for Zygoptera, but not for Anisoptera diversity (Table 2). The other five local variables (water depth, water diameter, bankside tree cover, bankside herb cover, and height of bankside vegetation) showed no significant correlation with species diversity (Table 2).

**Table 2:** Pearson correlation coefficients between local variables and species diversity (Shannon), with corresponding statistics (df = 9).

	Odonata		Zygoptera		Anisoptera	
	r	p	r	p	r	p
Water diameter (m)	−0.23	0.49	−0.4	0.22	−0.14	0.68
Water depth (cm)	−0.04	0.92	−0.46	0.16	0.02	0.96
Water surface cover (%)	−0.73	0.01	−0.66	0.03	−0.4	0.22
Bankside tree cover (%)	−0.39	0.23	−0.44	0.18	−0.05	0.88
Bankside herb cover (%)	0.06	0.85	−0.22	0.51	0.08	0.81
Plant height (cm)	0.1	0.77	−0.11	0.76	−0.09	0.78

Results regarding other diversity indices had the same outcome as detailed above (Supplementary Material, Table S2). For rarefied species richness the only significant local variable was the water surface cover for Zygoptera. In the case of Simpson diversity, the only significant local variable was again the water surface cover for Zygoptera and Odonata. Based on the Pielou’s evenness the only significant relationship was found again between the water surface cover and Odonata.

### Landscape variables

The landscape diversity increased from small scale (0.91 ± 0.21) towards intermediate (0.97 ± 0.23) and large scales (1.11 ± 0.19). The total length of watercourses (km) within the landscape also showed an increasing trend (small scale: 5.06 ± 2.57 km, intermediate scale: 13.83 ± 6.85, large scale: 42.97 ± 14.98). The forest patch proportion increased from large (9.85 ± 5.67) to middle (12.96 ± 13.13) and small scale (15.87 ± 13.91). The farmland patch size (ha) decreased only from large (25.06 ± 11.31) to middle (15.87 ± 7.25) and small scale (14.89 ± 7.95). The mean distance to the nearest forest patch was 174.46 (±253.45) meters.

Two variables showed significant correlation with Odonata diversity from the five tested variables on landscape scale. The total length of watercourses at the largest scale showed significant positive correlation with the diversity of Anisoptera (Table 3). The correlation was not significant for Zygoptera (Table 3), nor for the whole Odonata assemblage (Table 3).

**Table 3:** Pearson correlation coefficients between landscape variables and species diversity (Shannon) with corresponding statistics (df = 9).

	Scale (km)	Odonata		Zygoptera		Anisoptera	
		r	p	r	p	r	p
Landscape diversity	5	−0.01	0.98	−0.17	0.62	0.14	0.68
	2.5	−0.28	0.41	−0.45	0.17	0.10	0.78
	1.25	−0.15	0.67	−0.47	0.15	0.01	0.97
Length of watercourses	5	0.41	0.21	−0.06	0.86	0.63	0.04
	2.5	0.23	0.50	−0.02	0.95	0.42	0.20
	1.25	0.19	0.57	0.05	0.88	0.27	0.43
Forest patch proportion	5	−0.22	0.51	−0.46	0.16	−0.01	0.99
	2.5	−0.40	0.23	−0.49	0.13	−0.16	0.64
	1.25	−0.55	0.08	−0.55	0.08	−0.24	0.47
Farmland patch size	5	−0.74	0.01	−0.45	0.16	−0.55	0.08
	2.5	−0.45	0.16	−0.14	0.68	−0.60	0.05
	1.25	−0.44	0.17	0.00	1.00	−0.72	0.01
Distance to the nearest forest patch	1.25	0.39	0.24	0.07	0.83	0.23	0.49

**Figure 1:**
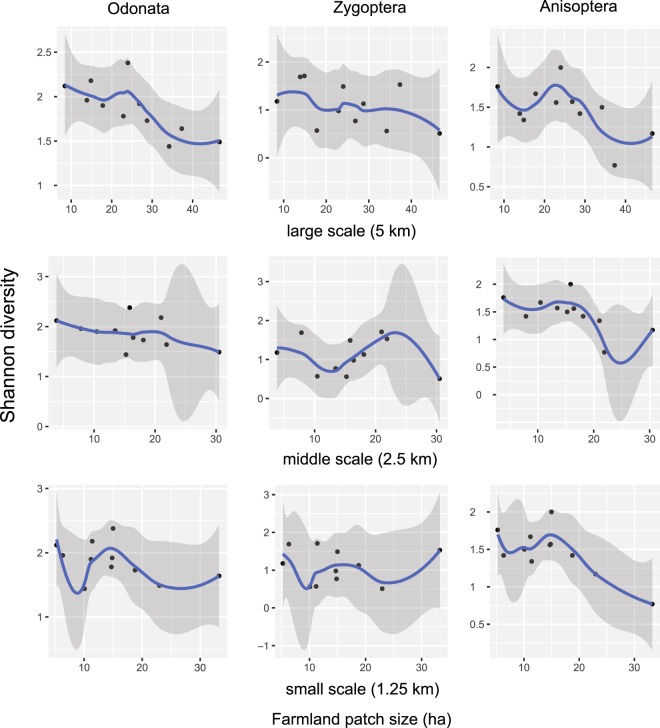
Relationship between farmland patch size and Odonata diversity at the studied landscape scales for the two suborders (Zygoptera and Anisoptera) and for the Odonata assemblages by locally weighted smoothed scatterplots (with 95% confidence interval around smooth – dark grey).

The farmland patch size showed significant negative correlations with species diversity at various landscape scales (Fig. 1). Correlation was significant for the whole Odonata diversity at the largest landscape scale (Table 3), although it was not significant for Zygoptera (Table 3), and only marginally significant for Anisoptera (Table 3). At the smaller landscape scale (radius = 2.5 km) correlation was only marginally significant but only for Anisoptera (Table 3). We found the same pattern at the smallest landscape scale (radius = 1.25 km), i.e. the correlation was significant for Anisoptera (Table 3).

At the smallest landscape scale (radius = 1.25 km) we also found a marginally significant correlation between the forest patch proportion and the diversity of Zygoptera (Table 3) and the diversity of whole Odonata (Table 3). Regarding the other landscape variables, no significant correlations were found (Table 3).

Results regarding other diversity indices than Shannon index showed the same relationships (Supplementary Material, Table S2) as detailed above. For rarefied species richness on landscape scale the only significant variables were the farmland patch size for Anisoptera and the forest patch proportion for Zygoptera. For Simpson diversity the only significant landscape scale variables were the farmland patch size for Anisoptera and Odonata, and the length of watercourses for Anisoptera. The Pielou’s evenness showed again significant relationship on landscape scale with the farmland patch size for Anisoptera and Odonata.

### Variable importance

The cover of emergent vegetation was the variable with the highest relative importance (both local (i.e. aquatic) and landscape-scale (terrestrial) in explaining the Odonata species diversity; it was followed by the farmland patch size on the 5 km scale (Table 4). The total length of watercourses on the 5 km scale had low relative importance, while the forest patch proportion on the 1.25 km scale had the lowest importance (Table 4).

**Table 4:** The analysed models (Gaussian errors) explaining species diversities (Shannon) of Odonata assemblages. AIC = Akaike’s information criteria. ω = Akaike weights. The “+” signs denote variables entered into the models.

Assemblage	Water surface cover	Total length of watercourses (5 km scale)	Forest patch proportion (1.25 km scale)	Farmland patch size (5 km scale)	AIC	ω
Odonata	+	+	+	+	−3.69	0.10
	+	+		+	−5.56	0.26
	+			+	−7.41	0.64
Anisoptera	+	+	+	+	7.70	0.08
	+		+	+	5.70	0.22
	+			+	4.24	0.46
	+				5.52	0.24
Zygoptera	+	+	+	+	13.56	0.14
	+	+		+	11.59	0.38
	+	+			12.22	0.28
	+				12.86	0.20
Relative importance	3.00	1.24	0.54	2.28		

## Discussion

The influence of water body attributes and the surrounding landscape on the Odonata assemblages along lowland watercourses were tested. The studied watercourses were the most stable aquatic environments from the whole hydrographic basin, because they persisted even in extreme dry summer periods. The short term stability of the aquatic ecosystems is of crucial importance for the breeding success and generational continuity of populations in most of species^27^. Furthermore, the quality of the terrestrial environment is also important for the populations because it provides habitats for mating, egg laying, feeding, resting and facilitates dispersal^28^. The results show that both local and landscape variables were important for the occurrence and abundance of Odonata. However, it is stressed that the two groups showed different sensitivities to the local and landscape variables.

Only one out of the six analysed local variables (the cover of emergent vegetation), had significant influence on the species richness and diversity of Odonata. Especially the Zygoptera showed significant sensitivity to this variable. Furthermore, Zygoptera diversity decreased with the increase of the cover of emergent vegetation. However, a relatively high cover of emergent vegetation on almost all sites was observed. A high open water surface cover may hamper the movement of Zygoptera, which has weaker flying and dispersal ability than the Anisoptera species^2^. Rouquette and Thompson^19^ report the importance of emergent vegetation in the case of *Coenagrion mercuriale*; it is underlined that high percentage of water surface cover is not favoured, at the same time open water positively affects the density of *C. mercuriale*.

In another study, where both Zygoptera and Anisoptera species were analysed from the perspective of water surface cover, Anisoptera species were more affected than Zygoptera species^18^. The explanation for the inconsistence between the findings and the previously cited results is regarded to water surface cover variability, namely that in this case the water surface cover was rather high which may have hindered the movement of Zygoptera. Most of the Zygoptera species are perchers, and detect intruders, or females sitting on different surfaces (plants, sticks) by watching around^2^. Shade of surveyed habitats had no significant effects on the Odonata assemblages, a result which is adverse to several reported relationships^29,30^. The findings regarding shade may be due to the relatively reduced shade in all selected habitats.

The relationship between the landscape variables and Odonata was significant only for the Anisoptera species, and this result supported the expectation. As expected from published literature, Anisoptera due to their higher dispersal ability are more sensitive to the landscape structure than Zygoptera^1,24,31^. This assumption was also confirmed in other studies, where it was reported that the more mobile Anisoptera were more sensitive to landscape variables at large scales, while Zygoptera were sensitive to local variables (i.e. water body related)^4,10^.

This difference in habitat sensitivity between the two Odonata suborders can explain the finding that total length of watercourses on a 5 km scale has a positive significant effect on Anisoptera diversity. Anisoptera have larger size, bigger muscle mass, and better thermoregulation than Zygoptera^32^, and thus better flying abilities. A longer watercourse network provides an extended habitat which provides more food, more oviposition site and more conspecific females, and higher survival chance. It was reported that in England the number of ponds in the surrounding area had no effect on species richness of dragonflies^28^. However, their largest spatial scale was of a 1600-meter-long radius, contrary to the 5 km long radius scale used in this study. In another study^4^ authors found that the distance to the nearest possible pond is a crucial factor in species occurrence: species richness decreased with increasing distance to the nearest suitable pond. In an experimental study where cattle tanks were used, the results show that both the distance to the nearest tank and the connectivity between artificial ponds affected significantly the species richness^15^. With increasing isolation, the dispersal between tanks decreased, and thus species richness declined.

The farmland patch size showed a significant negative effect on Odonata species diversity at a large scale (5 km), and on Anisoptera species diversity at the small (1.25 km) scale. The trend was the same for Anisoptera at the middle scale (2.5 km). The farmland patch size alludes to landscape fragmentation: increasing patch size results in landscape homogenization, with fewer buffer strips, bushes, forest patches, and presumably high fertilizer input. In the agriculturally intensified landscapes this means less space for maturation, feeding, and resting for the dragonflies.

The negative effects of the intensified land use on a large number of Odonata was presented by Ott^33^. A similar effect on Odonata species richness was described in another study where the species richness increased with larger areas of land under Higher Level Scheme^28^. The Higher Level Scheme, an agri-environmental scheme included pond-specific options that could potentially beneficial for Odonata, by assuring buffering in-field ponds in improved grassland or farmland, maintenance of high quality ponds, and pond creation and restoration.

Habitat structure and landscape configuration effect on species diversity was demonstrated in a study (Georgian Bay region, Canada). They showed that the habitat structure and other landscape variables calculated at increasing scales (1, 2, 4 and 8 km) was more important than boating pressure both for adults and for larvae^34^. In a study of the threatened dragonfly species *Sympetrum depressiusculum* Dolný *et al*.^35^ and Hykel *et al*.^36^ suggest that the heterogeneous terrestrial habitat structure is essential for the development of juveniles, and movement of adults, which preferred habitat patches with abundant vegetation. When the importance of land cover types per se and landcover heterogeneity was studied, authors showed that from nine land cover types, farmland percentage had positive effect on 9 species, and negative effect on 31 species. They also found that in the case of 73 species abundance increased with the increasing of landcover heterogeneity^21^. The landscape composition surrounding habitats was found to be one of the main determinants of Odonata diversity^17^. Habitat heterogeneity can be considered as the main reason for increasing species richness and diversity and has stronger impact than habitat size alone^37^. For some Odonata species the landscape heterogeneity is of great importance, while for others seems to be of less^38^.

Species diversity of Zygoptera showed a marginally significant decreasing trend with the increasing forest patch proportion in the surrounding habitat at the small scale (1.25 km). This relationship was underlined in a study where Odonata species richness decreased with increasing amounts of forest, especially on a 200 m scale^28^. Although the role of forests for Odonata has a voluminous literature^5,14,39^ the results showed that for the lowland Zygoptera species the increased amount of woodland could be an obstructive factor.

It was demonstrated that Odonata show different responses to local and landscape variables. While the Zygoptera species were mostly affected by local variables, the Anisoptera species were more sensitivity to landscape variables. This study further highlighted the need for simultaneous consideration of local (aquatic habitat) and landscape variables to understand fully the habitat use of Odonata. The findings suggest that an extensive land use management is necessary for a successful conservation management of Odonata assemblages. This kind of management supports species-rich Odonata assemblages and may also be beneficial for several other taxa such as amphibians, butterflies and farmland birds^40–42^. For preservation of species-rich Odonata assemblages future policies should take the landscape context into consideration and management actions should be directed toward regions where availability of extensively used areas is high.

## Material and Methods

### Sampling sites

**Figure 2:**
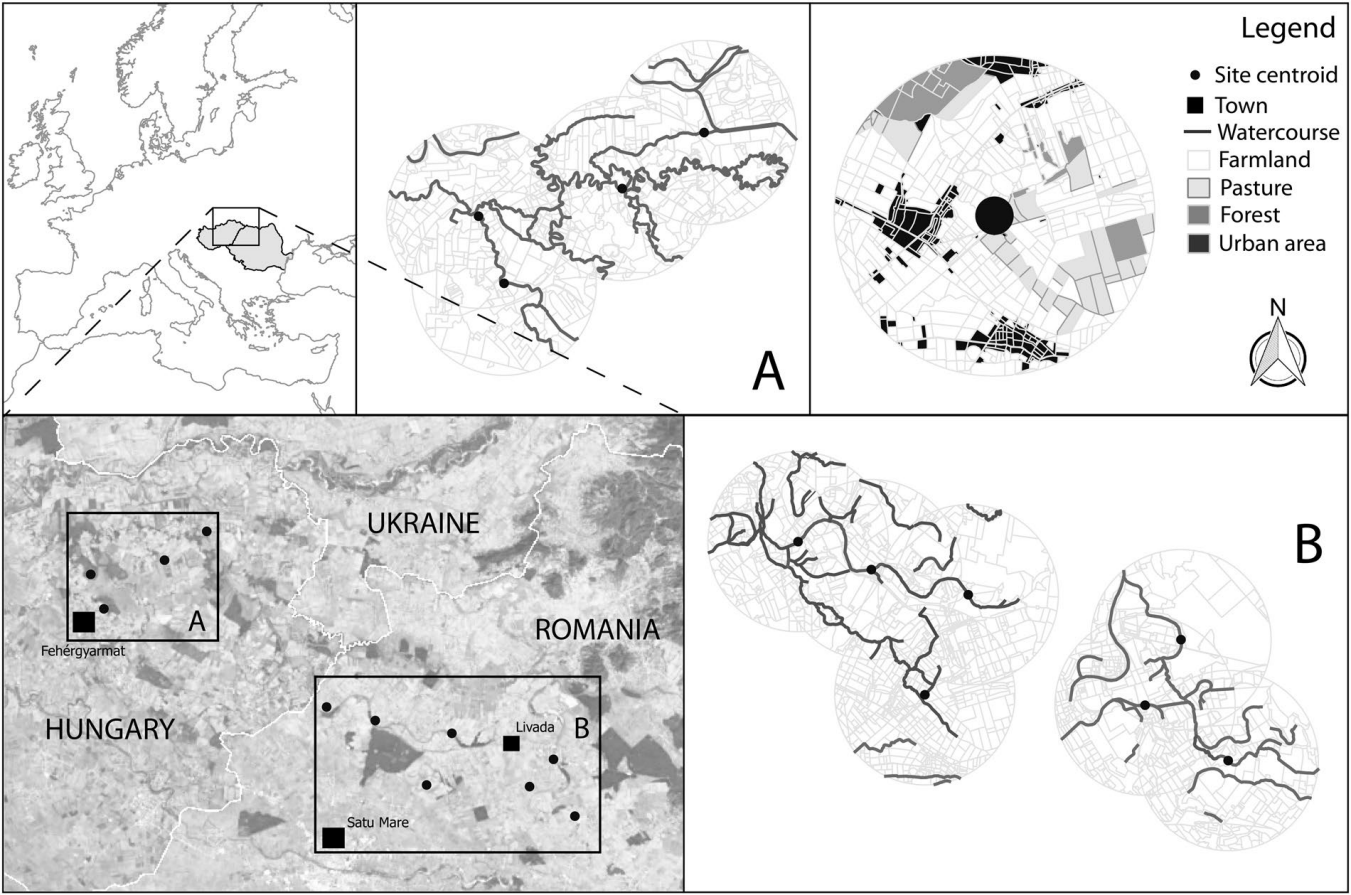
Study sites and their landscape neighbourhood. The sampled watercourse segments were positioned at the site centroids. The predominant cover types were farmlands, urban areas, forest patches, and pastures. Cover type boundaries were manually digitised by the authors. The digitised areas were acquired from Google Earth™ (http://earth.google.com/; © 2016 Google; © 2016 Geoeye; © 2016 DigitalGlobe). Maps were constructed in Quantum GIS (version 2.14.11 “Essen”; https://qgis.org/downloads/QGIS-OSGeo4W-2.14.11-1-Setup-x86_64.exe).

Lowland watercourses and their neighbouring habitats of North East Hungary (Szatmári Plain) and North West Romania (North-Partium) were surveyed in 2015 and 2016 (Fig. 2). The region surveyed in Hungary had cca. 62 km^2^ and that of Romania had 127 km^2^. The two survey years were dry^43^; in this context, it was identified in the studied area all the possible watercourses with substantial amount of water in order to implement our sampling design, and selected 11 study sites (4 in Hungary and 7 in Romania). The chosen watercourses were characterised by the presence of *Carex* sp., *Glyceria maxima*, *Mentha aquatica*, *Nuphar lutea*, *Sparganium erectum*, *Stratiotes aloides*, *Phragmites australis*, *Typha latifolia*, *T. angustifolia*. Banksides had rich herbaceous vegetation, with scarce shrub and tree cover. Watercourses were usually exposed to direct sunlight. Surveyed watercourses were at different distances from forest patches. All watercourses were at least on one bankside adjacent to agricultural fields, mostly farmlands. All surveyed watercourses were outside of urban areas. These watercourses are characterized by a slow water flow, with abundant still zones. The great majority of species are developing in these watercourses, even the species which require still water; thus, there are no vagrant species here or their occurrence is very unlikely.

### Data collection

The Odonata assemblages were sampled using a 500-meter-long transect along each watercourse. The sampling events dated from May to September, once a month, in warm, sunny days when the minimum temperature exceeded 20 °C, with wind speed under 15 km/h, and no considerable cloud cover were observed. The same person, walking at a steady pace counted every observed specimen in every sample event.

Every specimen was identified to species level. Species were identified both either visually or were caught with an insect net when visual identification was not possible on the site.

### Local and landscape variables

Local variables were recorded in six points across 500 m transect, for each studied watercourse. These following local variables were registered: water depth (meter, m), water width (m), water surface macrovegetation cover (percentage cover), bankside cover with trees and bushes (percentage cover), bankside cover with herbs (percentage cover), and average height of the bankside vegetation (cm). These variables were measured or estimated by the same person at every single sample event. The bankside cover with trees and bushes, and the average height of the bankside vegetation incorporates the shade characteristic of the habitats. Local abiotic variables were measured once at every sample site. These abiotic variables include: air temperature, wind speed, humidity, and the distance of visibility.

Landscape level variables were recorded in a circle with radii 1.25 (small scale), 2.5 (intermediate scale) and 5 km (large scale) around the midpoint of the sampled transects. These areas were digitised from the highest spatial resolution satellite images possible, acquired from Google Earth™ (http://earth.google.com/; © 2016 Google; © 2016 Geoeye; © 2016 DigitalGlobe). The maps were constructed from manually digitised cover type boundaries at a resolution ratio of 1:250 in Quantum GIS (version 2.14.11 “Essen”; Quantum GIS Development Team 2016). Cover types were delimited as farmland, pastures, orchards, urban areas, broad-leaved forests, bushy areas, embankments, dry riverbeds, rivers and lakes. The area (ha) of each cover type was calculated using Quantum GIS.

From the digitised maps the following variables were calculated: landscape diversity with Shannon index, total length of watercourses, proportion of forest patches, farmland patch size, and the distance to the nearest forest patch. The patch sizes of farmlands were used instead of their proportion in the landscape because the type of land use can be determined by the mean patch size of the crops in the landscape. All variables were calculated at all used scales, except the distance to the nearest forest patch, which was measured only in the smallest (1.25 km radius) circles. Total length of watercourses contained length of creeks and rivers. Landscape diversity, forest patch proportion, and farmland patch size, were calculated using the package LecoS^44,45^ in Quantum GIS.

### Statistical methods

The R programming language was used during the statistical calculations (R Development Core Team, version 3.5.0 2018). To assess Odonata assemblage characteristics Shannon diversity was calculated for each sampling site using function’diversity’ from package’vegan’^46^. We calculated rarefied species richness^46^, Shannon diversity, Simpson diversity and Pielou’s evenness^46^ for Anisoptera, Zygoptera and all Odonata. Then Goodness of Fit (GOF) tests were utilised to verify the normality assumption for each analysed outcome variable. Collinearity between the explanatory variables was assessed with Pearson correlation; no collinearity was detected (r < 0.5); therefore, we used all variables in the modelling^47^. For assessing correlations between local or landscape level environmental variables and assemblage diversities, Pearson’s correlation coefficients were calculated. Environmental variables that showed significant or marginally significant correlations were used to build linear models with Gaussian error distributions (using function ‘lm’). Backward stepwise selection procedure (using function ‘update’) were used selecting important variables. From all models, AICs and Akaike weights were calculated (ω) (using function ‘akaike.weights’ from package ‘qpcR’). Based on ω values, relative importance was calculated from the used environmental variable sets as described in Rhodes *et al*.^47^.

## Supplementary information

 Supplementary material Table S1.

Supplementary material Table S2.
