# Supplementary material for: Landscape-scale terrestrial factors are also vital in shaping Odonata assemblages of watercourses
Source: Sci Rep. 2019 Dec 3;9:18196. doi: 10.1038/s41598-019-54628-7 (PMC6890666; doi:10.1038/s41598-019-54628-7)
Supplement: Supplementary file 1 — Supplementary material Table S1. [file 41598_2019_54628_MOESM1_ESM.pdf]

Title:

Landscape-scale terrestrial factors are also vital in shaping Odonata assemblages of watercourses

Authors:

H. Beáta NAGY[1], Zoltán LÁSZLÓ[2*], Flóra SZABÓ[2], Lilla SZŐCS[2], György DÉVAI[3], Béla TÓTHMÉRÉSZ[1, 4]

Affiliations

[1]MTA-DE Biodiversity and Ecosystem Services Research Group, Debrecen, Egyetem sq. 1, H-4032 Hungary

[2]Hungarian Department of Biology and Ecology, Babeş-Bolyai University, str. Clinicilor nr. 5–7, 400006 Cluj-Napoca, Romania

[3]Department of Hydrobiology, University of Debrecen, Debrecen, Egyetem sq. 1, H-4032 Hungary

[4]Ecology Department, University of Debrecen, Debrecen, Egyetem sq. 1, H-4032 Hungary

*Corresponding author: E-mail address: laszlozoltan@gmail.com, ORCID: http://orcid.org/0000-0001-5064-4785.

Table S1. Encountered specimen sums from years 2015, 2016 at each study site with site coordinates.

Species	Suborder	Site 1	Site 2	Site 3	Site 4	Site 5	Site 6	Site 7	Site 8	Site 9	Site 10	Site 11	Total
Chalcolestes parvidens	Zygoptera	3	99	0	0	12	2	0	5	1	3	0	125
Lestes barbarus	Zygoptera	0	219	2	12	0	0	0	0	0	9	1	243
Lestes dryas	Zygoptera	0	0	0	0	0	0	0	0	0	0	0	0
Lestes sponsa	Zygoptera	48	0	2	0	0	0	0	0	0	0	0	50
Sympecma fusca	Zygoptera	33	3	0	4	1	0	0	0	2	0	0	43
Calopteryx splendens	Zygoptera	2	52	107	4	59	5	21	22	1	3	71	347
Platycnemis pennipes	Zygoptera	2	30	122	2	78	0	10	3	8	1	386	642
Coenagrion puella	Zygoptera	263	180	171	8	283	127	46	601	20	323	846	2868
Coenagrion pulchellum	Zygoptera	31	9	59	0	6	20	1	53	437	3	10	629
Erythromma najas	Zygoptera	0	0	0	0	0	0	0	0	9	0	4	13
Erythromma viridulum	Zygoptera	0	0	0	0	0	0	0	0	3	0	0	3
Ischnura elegans	Zygoptera	10	2	165	14	36	3	0	9	5	1	41	286
Ischnura pumilio	Zygoptera	6	0	35	24	140	4	2	1	8	5	1	226
Pyrrhosoma nymphula	Zygoptera	0	4	0	0	0	0	1	0	0	11	0	16
Aeshna affinis	Anisoptera	20	81	12	58	3	18	50	34	93	26	35	430
Aeshna cyanea	Anisoptera	0	1	0	0	15	0	2	0	0	2	0	20
Aeshna mixta	Anisoptera	5	3	3	0	0	2	0	11	33	0	5	62
Anaciaeschna isoceles	Anisoptera	30	2	7	1	19	2	1	15	177	2	99	355
Anax imperator	Anisoptera	7	0	4	2	0	0	1	2	2	0	1	19
Brachytron pratense	Anisoptera	23	12	0	5	31	3	2	0	8	3	6	93
Onychogomphus forcipatus	Anisoptera	0	0	0	0	0	0	0	0	1	0	0	1
Somatochlora flavomaculata	Anisoptera	0	53	4	0	26	22	101	0	5	0	0	211
Somatochlora meridionalis	Anisoptera	0	0	0	0	2	0	0	0	0	0	2	4
Libellula depressa	Anisoptera	14	0	8	3	107	2	6	7	0	20	4	171
Libellula fulva	Anisoptera	3	68	70	0	64	34	89	6	3	0	109	446
Libellula quadrimaculata	Anisoptera	0	0	0	0	0	0	0	0	0	0	1	1
Orthetrum albistylum	Anisoptera	0	0	2	2	0	0	0	0	0	0	0	4
Orthetrum brunneum	Anisoptera	0	0	0	0	0	0	0	3	0	0	0	3
Orthetrum coerulescens	Anisoptera	1	0	0	0	0	5	1	0	0	0	3	10
Sympetrum flaveolum	Anisoptera	0	0	0	18	0	0	5	0	0	0	0	23
Sympetrum meridionale	Anisoptera	90	22	0	70	0	0	0	0	0	8	16	206
Sympetrum sanguineum	Anisoptera	105	1049	112	123	107	116	365	142	287	191	307	2904
Sympetrum striolatum	Anisoptera	0	0	0	0	17	31	52	66	51	41	26	284
Sympetrum vulgatum	Anisoptera	5	0	0	0	46	0	0	2	88	0	5	146
	Country	RO	RO	RO	RO	RO	RO	RO	HU	HU	HU	HU	
	E	22,97974	23,01215	23,14433	22,93974	23,20644	22,88498	23,17116	22,51728	22,68234	22,53596	22,62234	
	N	47,83253	47,8827	47,82729	47,89531	47,79951	47,90931	47,86018	48,03023	48,07118	47,99738	48,04377	
