# Supplementary material for: Landscape-scale terrestrial factors are also vital in shaping Odonata assemblages of watercourses
Source: Sci Rep. 2019 Dec 3;9:18196. doi: 10.1038/s41598-019-54628-7 (PMC6890666; doi:10.1038/s41598-019-54628-7)
Supplement: Supplementary file 2 — Supplementary material Table S2. [file 41598_2019_54628_MOESM2_ESM.pdf]

Title:

Landscape-scale terrestrial factors are also vital in shaping Odonata assemblages of watercourses

Authors:

H. Beáta NAGY[1], Zoltán LÁSZLÓ[2*], Flóra SZABÓ[2], Lilla SZŐCS[2], György DÉVAI[3], Béla TÓTHMÉRÉSZ[1, 4]

Affiliations

[1]MTA-DE Biodiversity and Ecosystem Services Research Group, Debrecen, Egyetem sq. 1, H-4032 Hungary

[2]Hungarian Department of Biology and Ecology, Babeş-Bolyai University, str. Clinicilor nr. 5–7, 400006 Cluj-Napoca, Romania

[3]Department of Hydrobiology, University of Debrecen, Debrecen, Egyetem sq. 1, H-4032 Hungary

[4]Ecology Department, University of Debrecen, Debrecen, Egyetem sq. 1, H-4032 Hungary

*Corresponding author: E-mail address: laszlozoltan@gmail.com, ORCID: http://orcid.org/0000-0001-5064-4785.

Table S2. Pearson correlation coefficients of localand landscape variables with species richness and diversity, with corresponding statistics (df = 9).

		Odonata		*Zygoptera*		*Anisoptera*	
		est.	p	est.	p	est	p
**Species richness**	*Water diameter (m)*	-0,27	0,41	-0,29	0,39	-0,38	0,25
	Water depth (cm)	0,00	1,00	-0,40	0,23	-0,29	0,38
	Water surface cover (%)	-0,54	0,09	***-0,62***	***0,04***	-0,38	0,25
	Bankside cover (%)	0,05	0,88	-0,06	0,86	-0,23	0,50
	Bankside tree cover (%)	-0,37	0,26	-0,57	0,06	-0,11	0,75
	Plant height (cm)	-0,21	0,54	-0,10	0,77	-0,32	0,34
**Shannon diversity**	*Water diameter (m)*	-0,23	0,49	-0,40	0,22	-0,14	0,68
	Water depth (cm)	-0,04	0,92	-0,46	0,16	0,02	0,96
	Water surface cover (%)	***-0,73***	***0,01***	***-0,66***	***0,03***	-0,40	0,22
	Bankside cover (%)	0,06	0,85	-0,22	0,51	0,08	0,81
	Bankside tree cover (%)	-0,39	0,23	-0,44	0,18	-0,05	0,88
	Plant height (cm)	0,10	0,77	-0,11	0,76	-0,09	0,78
**Simpson diversity**	*Water diameter (m)*	-0,17	0,61	-0,47	0,14	-0,10	0,77
	Water depth (cm)	0,07	0,84	-0,49	0,12	0,02	0,95
	Water surface cover (%)	***-0,62***	***0,04***	***-0,62***	***0,04***	-0,37	0,26
	Bankside cover (%)	0,25	0,47	-0,23	0,49	0,17	0,62
	Bankside tree cover (%)	-0,48	0,13	-0,39	0,23	-0,16	0,63
	Plant height (cm)	0,29	0,39	-0,11	0,75	-0,01	0,98
**Evenness**	*Water diameter (m)*	-0,30	0,38	-0,45	0,16	-0,12	0,73
	Water depth (cm)	-0,03	0,93	-0,50	0,12	0,11	0,76
	Water surface cover (%)	***-0,73***	***0,01***	***-0,60***	***0,05***	-0,47	0,14
	Bankside cover (%)	0,14	0,67	-0,11	0,75	0,12	0,73
	Bankside tree cover (%)	-0,44	0,18	-0,44	0,18	-0,11	0,75
	Plant height (cm)	0,15	0,65	-0,02	0,95	-0,09	0,78

			Odonata		*Zygoptera*		*Anisoptera*	
		Scale (km)	r	p	r	p	r	p
**Species richness**	*Landscape diversity(Shannon)*	5,00	-0,06	0,86	-0,28	0,40	0,50	0,12
		2,50	-0,20	0,56	-0,54	0,09	0,48	0,13
		1,25	-0,26	0,44	-0,55	0,08	0,24	0,49
	Length of watercourses (km)	5,00	0,15	0,65	-0,16	0,64	0,57	0,07
		2,50	-0,18	0,59	-0,21	0,53	0,18	0,60
		1,25	-0,25	0,46	-0,21	0,53	0,26	0,45
	Forest patch proportion (%)	5,00	-0,02	0,95	-0,39	0,23	0,20	0,56
		2,50	-0,26	0,45	-0,50	0,12	0,10	0,77
		1,25	-0,57	0,07	***-0,69***	***0,02***	-0,05	0,89
	Farmland patch size (ha)	5,00	-0,52	0,10	-0,51	0,11	-0,40	0,22
		2,50	-0,53	0,09	-0,29	0,39	-0,39	0,23
		1,25	-0,49	0,13	-0,15	0,67	***-0,62***	***0,04***
	Dist. to the near. forest patch (km)	1,25	0,55	0,08	0,23	0,50	0,45	0,17
**Shannon diversity**	*Landscape diversity(Shannon)*	5,00	-0,01	0,98	-0,17	0,62	0,14	0,68
		2,50	-0,28	0,41	-0,45	0,17	0,10	0,78
		1,25	-0,15	0,67	-0,47	0,15	0,01	0,97
	Length of watercourses (km)	5,00	0,41	0,21	-0,06	0,86	***0,63***	***0,04***
		2,50	0,23	0,50	-0,02	0,95	0,42	0,20
		1,25	0,19	0,57	0,05	0,88	0,27	0,43
	Forest patch proportion (%)	5,00	-0,22	0,51	-0,46	0,16	-0,01	0,99
		2,50	-0,40	0,23	-0,49	0,13	-0,16	0,64
		1,25	-0,55	0,08	-0,55	0,08	-0,24	0,47
	Farmland patch size (ha)	5,00	***-0,74***	***0,01***	-0,45	0,16	-0,55	0,08
		2,50	-0,45	0,16	-0,14	0,68	-0,60	0,05
		1,25	-0,44	0,17	0,00	1,00	***-0,72***	***0,01***
	Dist. to the near. forest patch (km)	1,25	0,39	0,24	0,07	0,83	0,23	0,49
**Simpson diversity**	*Landscape diversity(Shannon)*	5,00	0,04	0,90	-0,13	0,70	0,15	0,65
		2,50	-0,20	0,56	-0,39	0,24	0,11	0,74
		1,25	-0,05	0,89	-0,45	0,17	0,03	0,92
	Length of watercourses (km)	5,00	0,48	0,14	-0,01	0,98	***0,63***	***0,04***
		2,50	0,31	0,35	0,06	0,86	0,42	0,20
		1,25	0,29	0,38	0,16	0,64	0,27	0,42
	Forest patch proportion (%)	5,00	-0,11	0,74	-0,44	0,18	-0,03	0,92
		2,50	-0,26	0,43	-0,44	0,18	-0,16	0,63
		1,25	-0,41	0,21	-0,48	0,14	-0,22	0,51
	Farmland patch size (ha)	5,00	***-0,76***	***0,01***	-0,42	0,20	***-0,62***	***0,04***
		2,50	-0,41	0,22	-0,12	0,73	***-0,63***	***0,04***
		1,25	-0,46	0,16	0,04	0,91	***-0,83***	***0,00***
	Dist. to the near. forest patch (km)	1,25	0,38	0,25	0,03	0,93	0,22	0,51
**Evenness**	*Landscape diversity(Shannon)*	5,00	0,02	0,95	-0,15	0,67	0,17	0,62
		2,50	-0,26	0,44	-0,40	0,22	0,08	0,82
		1,25	-0,09	0,79	-0,42	0,20	0,06	0,86
	Length of watercourses (km)	5,00	0,33	0,32	-0,04	0,90	0,54	0,09
		2,50	0,06	0,86	-0,02	0,95	0,21	0,53
		1,25	0,07	0,85	0,07	0,84	0,05	0,87
	Forest patch proportion (%)	5,00	-0,24	0,48	-0,46	0,15	0,01	0,99
		2,50	-0,39	0,24	-0,46	0,16	-0,17	0,62
		1,25	-0,55	0,08	-0,50	0,12	-0,29	0,39
	Farmland patch size (ha)	5,00	***-0,69***	***0,02***	-0,43	0,18	-0,52	0,10
		2,50	-0,39	0,24	-0,14	0,68	-0,55	0,08
		1,25	-0,42	0,20	-0,02	0,95	***-0,75***	***0,01***
	Dist. to the near. forest patch (km)	1,25	0,39	0,24	0,03	0,93	0,29	0,38
